# Recent Advances in Hydroxyapatite Scaffolds Containing Mesenchymal Stem Cells

**DOI:** 10.1155/2015/305217

**Published:** 2015-05-28

**Authors:** John Michel, Matthew Penna, Juan Kochen, Herman Cheung

**Affiliations:** ^1^Department of Biomedical Engineering, College of Engineering, University of Miami, 1251 Memorial Drive, MEA 219A, Coral Gables, FL 33146, USA; ^2^Geriatric Research, Education and Clinical Center (GRECC), Miami Veterans Affairs Medical Center, 1201 NW 16th Street, Miami, FL 33125, USA

## Abstract

Modern day tissue engineering and cellular therapies have gravitated toward using stem cells with scaffolds as a dynamic modality to aid in differentiation and tissue regeneration. Mesenchymal stem cells (MSCs) are one of the most studied stem cells used in combination with scaffolds. These cells differentiate along the osteogenic lineage when seeded on hydroxyapatite containing scaffolds and can be used as a therapeutic option to regenerate various tissues. In recent years, the combination of hydroxyapatite and natural or synthetic polymers has been studied extensively. Due to the interest in these scaffolds, this review will cover the wide range of hydroxyapatite containing scaffolds used with MSCs for *in vitro* and *in vivo* experiments. Further, in order to maintain a progressive scope of the field this review article will only focus on literature utilizing adult human derived MSCs (hMSCs) published in the last three years.

## 1. Introduction

Bone related traumas and lesions are painful conditions that affect millions of people on a daily basis. Further, the frequency of these conditions is bound to increase in the world population in coming years especially due to increased life expectancy. The voluminous number of cases that arise on a yearly basis warrants the search for new, practical constructs that can efficiently replace bone, effectively treating these ailments. Additionally, the high frequency of cases that will arise in the future will make current methods of treatment unavailable to a great majority, thus making a substitute construct the primary method of care.

Current solutions to many of these issues include replacement of damaged bone tissue with either autologous—tissue originating from the patient—or allogeneic—tissue originating from another person—bone grafts. Autologous grafts are currently held as the gold standard of the field but bring with them a series of burdens that may not be manageable in most patients [1, 2]. First, bone graft sources are not always readily available from these patients, since many patients who need bone grafts usually are not eligible for autologous grafts (i.e., osteoporosis patients have systemic, rather than local, bone degeneration making grafting another bone difficult). Second, large lesions in bone are not reparable solely by autologous grafting because of the limited supply of bone that can be grafted from each patient [1, 2]. Third, grafting bone from the patient also results in increased pain and morbidity [1, 2]. Allogeneic sources (mainly cadaveric sources), conversely, bring with them concerns of immunogenic response and consequent tissue rejection, which decreases the chance of host integration [1]. Additionally, allogeneic sources will become scarcer as a higher percentage of the population begins to express the aforementioned pathologies and require an allogeneic graft.

The rising difficulties of the previous conditions have led the field to look elsewhere for solutions. Tissue engineering of bone and cartilage has become an attractive solution due to the ability to control the parameters of the designed constructs and tailor them to the native characteristics of the diseased area. Optimizing the parameters of designed constructs gives these grafts the potential of being more efficient than autologous/allogeneic sources. The traditional tissue engineering approach entails using a biomaterial-cell combinatorial approach. The biomaterial is used as a scaffold to match the bulk properties of the tissue as well as to ensure proper cell-matrix cross-talk, housing the seeded cells and giving them the proper signals to maintain their phenotypic properties. It should also be nontoxic, nonimmunogenic, and biocompatible [3]. Cells are added for environment remodeling and regeneration and act as the dynamic aspect of the biomaterial-cell combination. These are added to the construct in hopes that they match the functionality of the native tissue, provide remodeling to the construct to aid in host integration, and/or are able to spur the host tissue to perform desired actions. Engineering a successful bone graft scaffold is usually based on four parameters: (i) it must act as a morphogenic signal for osteoinduction, (ii) it must have responsive host cells that can receive and respond to the morphogenic signals, (iii) it must serve as a scaffold onto which the seeded cells can grow and remodel, and (iv) it must be placed close to a viable host bed of vasculature [4]. With regard to osteobiologics, the engineered construct must match both the functionality and bulk properties of the native tissue. Additionally, the engineered construct must also be modifiable by both seeded cells and host cell populations; this ensures osteoconduction, host integration, osteoinduction of the cells in the scaffold, and the capacity for remodeling by the host or graft cells [1].

The field of tissue engineering has gravitated towards using undifferentiated stem cells as the best option for engineered constructs [3]. Stem cells are cells that have some degree of inherent potency, meaning they have the potential to differentiate into various cell types; their degree of potency can range from totipotent, enabling them to differentiate into any cell type including embryonic tissues, to multi-/unipotent, where they can only differentiate into one or a few cell types. Stem cells have been extensively used in tissue engineering due to their easy expandability in culture, their versatility of use based on their multi-/pluripotent characteristics, and their ability to dictate changes in their surrounding environment via cytokines and release of growth factors [5]. Although there seems to be many stem cell options to choose from in terms of potency, clinically relevant scaffolds clearly favor multipotent adult stem cells over pluripotent embryonic stem cells (ESCs) and induced pluripotent stem cells (iPSCs) [5]. Although the two latter have more versatility in terms of cell fates, long-term self-renewal, and sustenance of pluripotency, they also represent a potential source of teratoma or neoplasm formation and immunological incompatibility [5]. Further, ESCs also bring with them legal and ethical issues, whereas iPSCs have epigenetic memory from past lineages that interfere with the induction into desired lineages [5].

Adult stem cells, specifically mesenchymal stem cells (MSCs), are found naturally in native tissues such as blood, adipose tissue, and trabecular bone, representing a natural source of cells from the patient [3]. Further, these cells lack expression of major histocompatibility complex I and costimulatory molecules like CD40, CD80, and CD86, which makes them largely nonimmunogenic [5]. Additionally, they are able to suppress the immune system via mechanisms that are still widely unknown; this makes the use of allogeneic MSCs largely immunoinert [5]. As additional support to using MSCs for bone engineered constructs, MSCs demonstrate a natural ability to regenerate specific bone cell types, largely facilitating the induction mechanism into osteogenic lineages while still having some short term sustenance of multipotency [5]. However, although they present many beneficial characteristics, long-term culture of MSCs yields either terminal differentiation or senescence due to telomere shortening [5].

Additionally, although MSCs are inducible to the bone lineage, the bone constructs must also match the mechanical properties of the native tissue in order to achieve the same level of functionality of the native tissue. For this reason, the field has turned to hydroxyapatite (HA), Ca_10_(OH)_2_(PO_4_)_6_, a naturally occurring mineral which is chemically similar to the inorganic minerals of the bone [4]. Due to the chemical similarities to the inorganic materials found in bone, HA has been proven to be both osteoconductive and osteoinductive, making it a good option for bone replacement scaffolds [4, 6]. Additionally, its chemical similarity to the inorganic bone matter makes it biocompatible, modifiable by the hosts' osteoclasts, and slowly biodegrading* in situ* [6]. It has also been proven that HA shows good integration with both soft and hard tissues, making it heavily used in both bone tissue engineering and orthopedic and dental implants [4]. Moreover, HA is a porous material which allows ingrowth of capillaries and other vessels; this results in the perfusion of metabolic oxygen and nutrients to cells that lie in the scaffold, as well as cells from the host that integrate into it [4]. Although it presents a series of favorable characteristics for bone regeneration, unmodified HA has low mechanical strength, making it useless for replacement of load bearing bones [4]. For this reason, HA must be paired with another material to keep the osteoconductive and inductive properties of the HA, while adopting the mechanical properties of the newly incorporated material.

As both MSCs and HA individually have favorable characteristics for osteodifferentiation and osteoregeneration, a multitude of studies in recent years has focused on using them in combination to achieve a bone-type construct that is capable of equating, or even surpassing, autologous bone grafting as a solution to bone lesions. The methods these studies use to improve the mechanical properties of HA scaffolds to match those of bone are worth investigating and are what give the HA based scaffold the possibility of being used as a complete bone graft in both load bearing and non-load bearing bones. The optimal characteristics of this combination give this technology exciting potential as a complete replacement of both autologous and allogeneic sources, having both more availability and less harm to the patient.

## 2. Relevant Bone Biology and Pathways

In order to gauge the osteogenic potential of these graphs through current metrics, one must first understand the pathways behind bone formation and degradation. In the body, bone can develop either through the process of intramembranous ossification in which mesenchymal tissue directly differentiates into bone or endochondral ossification in which mesenchymal tissue first differentiates into cartilage before further ossification [7]. Additionally, bone is not a static tissue as it is constantly remodeled by the competing actions of bone forming osteoblasts and bone depleting osteocytes. Due to the complexity of these processes, tight coordination between osteo-, chondro-, and vasculogenic differentiation is required for the proper formation of bone [8].

The differentiation of MSCs into osteoblasts is largely controlled by the TGF-*β*, BMP, Smad and p38 MAPK, and Runx2 signaling pathways. These osteoblasts then begin the process of new bone formation by secreting the osteoid, which is the organic phase of bone and consists of a dense type I collagen network that is infused with osteocalcin and osteopontin. Osteoids then undergo the process of mineralization in which HA crystals are deposited and the bone becomes more rigid; alkaline phosphatase (ALP) activity can be detected at this stage of differentiation [9]. Therefore, increases in the aforementioned genes, proteins, and growth factors can be considered indicators of osteogenic differentiation.

Furthermore, when aiming to regenerate bone tissue it is often necessary to consider the surrounding and supporting tissues which are cartilage and vasculature, respectively. Cartilage is composed of negatively charged proteoglycans (such as glycosaminoglycan) and type II collagen, which creates structure much more flexible than bone [10]. Due to the proximity between cartilage and bone, issues in the bone will also have negative repercussions in cartilage tissue. For example, when aggressive neoplasms form in bone tissue the following resection may involve the partial removal of cartilage. One may wish to differentiate cartilaginous and osteogenic tissue in the same scaffold in order to replace the resected tissue. Therefore, although chondrogenic differentiation is still not well understood it is important to touch upon some of the defining features of it. The expression of Sox9 and the BMP and TGF*β* pathways both correspond to this differentiation [10]. Additionally, when one aims to create functional bone tissue for* in vivo* use it is necessary to vascularize the tissue. In order to determine whether an increase in differentiation towards a vasculature lineage has occurred one must assay for factors common in these tissues. Factors which can be used to test for angiogenesis include endothelial cell markers CD31, von vW, and vascular endothelial growth factor (VEGF) [11].

## 3. Hydroxyapatite Composite Scaffolds

**Figure 1:**
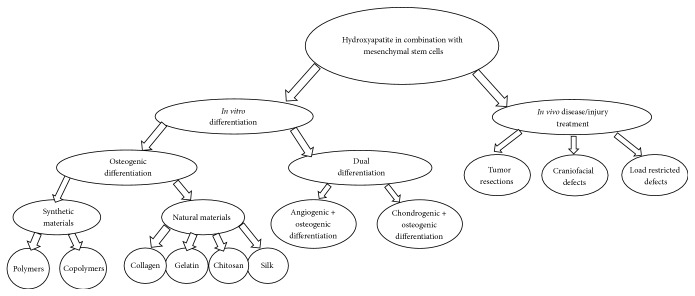
Graphic scheme of the categorization of topics in this review. For the purposes of this review, if materials were more than 50% natural they were considered natural or vice versa.

In the pursuit of using hydroxyapatite containing scaffolds for regenerative purposes, the choice of supporting material is often paramount. One may choose to combine HA with another material for a multitude of reasons like improved strength, increased porosity, altered cell binding abilities, and so forth [42]. In this line of thought, a great variety of papers have been dedicated to the characterization of scaffolds synthesized via the combinatorial approach of HA with another supporting material [43]. Due to the breadth of materials used in the literature for the treatment of specific osteodiseases, a background into the materials is often necessary. Therefore, this review will not only cover how HA containing scaffolds have been used to treat defects, but also introduce the materials used in terms of their osteogenic potential. We present Figure 1 representing how the information in this review is categorized.

## 4. *In Vitro* Differentiation

### 4.1. Osteogenic Differentiation* In Vitro*

#### 4.1.1. Natural Materials in Combination with Hydroxyapatite

Natural materials tend to have good cellular adhesion and remodeling properties but can also carry a high risk of immune response. These include collagen, gelatin, and fibrinogen. Synthetic materials, however, are less immunogenic and more customizable but carry higher risks of toxicity [44]. A summary of the literature involving natural materials in combination with HA for the following sections can be found in Table 1. 

*(1) Collagen*. Collagen is the one of the most studied natural polymers due to its biodegradability, biocompatibility, and porosity. However, collagen has a lack of rigidity which makes its use difficult in cases where scaffolds must be load bearing [45]. Collagen can be strengthened by the addition of other materials which was the case in a recent study conducted by Antebi et al. where HA was used to strengthen collagen through a polymer-induced liquid precursor (PILP) in combination with dynamic flow conditions. Briefly, PILPs are complexes which form when molecules capable of binding to calcium and phosphate (polyaspartic acid) do so in aqueous environments. PILPs infiltrate collagen scaffolds uniformly and deposit calcium and phosphate inside the fibrils, which crystallize into HA. This method was used to produce porous collagen/HA scaffolds which were subsequently coated with fibronectin and seeded with MSCs. Fibronectin was not seen to influence the attachment of cells and collagen/HA scaffolds showed better MSC infiltration. Although infiltration into the collagen/HA scaffolds by MSCs is demonstrated, additional quantification of the staining results and further osteoblast staining are not shown. Therefore, these further tests are required to conclusively show osteogenic differentiation [12].

In a study by Weszl et al. the coating of allographs versus HA (BioOss) using fibronectin, collagen, and albumin was compared and contrasted for MSC and dental pulp stem cell (DPSC) attachment. It was seen that only albumin coating improved MSC and DPSC attachment for allographs, but no coating changed the attachment of either cell type for HA scaffolds. Furthermore, the use of a rotating bioreactor to create dynamic culture conditions was seen to be superior to culturing in static conditions for both cell types. Therefore, the allograph was seen to be superior to HA throughout this experiment indicating that HA has not yet surpassed allographs in terms of bone regeneration [13].

*(2) Gelatin*. Another often used natural polymer is gelatin, which is the denatured version of collagen. Gelatin/HA scaffolds were considered for their potential in bone regeneration by Rungsiyanont et al. after the seeding of MSCs, human periodontal ligament (PDL) fibroblasts, and primary cells from hip bones (HBCs). Coprecipitation was used to create scaffolds with gelatin/HA percentages of 2.5%/2.5% and 2.5%/5%. Alkaline phosphatase (ALP) expression determined that MSCs osteoblast activity and by extension osteogenic differentiation were higher for the scaffolds with lower concentration of hydroxyapatite. Both hydroxyapatite scaffolds had higher ALP expression than controls and scanning electron microscopy (SEM) images showed good attachment and growth on both scaffolds for the MSCs. They concluded, therefore, that the use of gelatin/HA scaffolds increased osteogenic differentiation; however, too much HA was seen to be detrimental [14]. In order to improve the mechanical properties of HA/gelatin scaffolds and create a material with similar properties to natural bone Barbani et al. included gellan gum in the HA/gelatin composite. The scaffolds were seeded with MSCs taken from the Wharton jelly of the umbilical cord. The authors reported that after 21 days in culture the MSCs grew favorably as determined by SEM and hematoxylin and eosin (H&E) staining. Although these results are encouraging the authors performed neither additional staining nor gene expression to determine the ability of the material to induce osteogenic differentiation. Additionally, MSCs were not seeded on control scaffolds such as HA/gelatin or gelatin. Although the results are promising more tests are required to determine the potential for bone regeneration [15].

*(3) Chitosan*. Chitosan (CS) is a polysaccharide that has been used as a composite with hydroxyapatite for repairing bone tissue [46]. Kim et al. used HA to increase the osteodifferentiation potential of chitosan. The chitosan/HA scaffolds were created through a coprecipitation reaction followed by a spinning procedure and were seeded with bone marrow-derived MSCs (BMSCs). As early as five days following seeding a higher proliferative potential of the composite scaffold was demonstrated in comparison to the chitosan only scaffold. When osteogenic medium was used in conjunction with scaffolds, osteodifferentiation activity was higher in composite scaffolds than pure chitosan scaffolds. Additionally, gene expression showed that osteocalcin activity was significantly higher throughout all time points and ALP, Col1*α*I, and Runx2 were seen to increase earlier and with greater magnitude in the chitosan scaffolds containing HA. Staining indicated that ALP activity followed a similar trend as ALP expression and osteocalcin staining followed the same trend as osteocalcin expression. The author attributes the results to a more osteogenic nature of the HA-chitosan scaffolds inducing more rapid proliferation and differentiation of MSCs [16].

The following papers review cases in which CS has been used in combination with nHA. Chen et al. who used MSCs seeded on a biopolymer polyelectrolyte complex from CS and hyaluronic acid showed the biocompatibility and bioactivity of their respective scaffold design. The biocompatibility was concluded through an assessment of the proliferation of the MSCs on the scaffold with an MTT assay, while the osteogenic activity was determined through the standard ALP activity [17]. Another HA containing chitosan scaffold to recently be studied was synthesized by Wang et al. In this study nanohydroxyapatite- (nHA-) chitosan scaffolds created through a freeze-drying method and modified by cold atmospheric plasma (CAP) treatment were characterized. CAP entails propelling cold atmospheric plasma at a scaffold to enhance surface properties [47]. When MSCs were exposed to osteogenic differentiation conditions and seeded on scaffolds with CAP treatment, a significant increase in protein synthesis, calcium deposition, and collagen content was observed in comparison to the untreated scaffolds. Furthermore, SEM imaging indicated better morphological features and deeper penetration of cells in the CAP modified scaffolds than in the control scaffolds. The author attributes the increase in surface hydrophilicity, porosity, roughness, fibronectin adsorption, and vitronectin adsorption (*P* < 0.1) to chitosan fraying during CAP treatment. Although this study did not compare the results to a chitosan control it fully demonstrates the use of CAP treatment as a potential surface modifier to increase the osteodifferentiative potential of scaffolds [18]. A unique approach is taken by Ambre et al. in using a mineralized HA synthesized with nanoclays in combination with chitosan/polygalacturonic acid (CS/PgA) to form a novel scaffold [19]. The results showed that mineralized nodules formed on the scaffold when MSCs were seeded in the absence of osteogenic additives as shown with Alizarin red staining (ARZ). The MSCs also showed differentiation toward osteogenic fate as confirmed with ALP activity; however, it is worth noting that the scaffold without the HA clay has greater ALP activity which the authors attributed to mineralization of the extracellular matrix.

*(4) Silk*. Silk has also been used as a scaffold for MSCs due to its high strength and biocompatibility [48]. In another study performed by Bhumiratana et al. silk/HA scaffolds were synthesized through the use of NaCl as a porogen. Scaffolds with different HA percentages were synthesized and seeded with MSCs. It was seen that using higher concentrations of HA initially retarded cell growth. However, micro-CT showed that scaffolds with higher concentrations of HA induced more mineralization and trabecular-like structure formation. The authors report a higher staining for collagen I, bone sialoprotein, and osteocalcin as well as higher calcium production for groups with higher percentages of HA. These results indicate that silk/HA scaffolds can promote osteogenic differentiation [20].

#### 4.1.2. Synthetic Materials in Combination with Hydroxyapatite

Although natural materials show great potential because of their accessibility and inborn biocompatibility, synthetic materials have a high level of control of their various properties. Some examples of synthetic materials are polylactic acid (PLA), polycaprolactone (PCL), and poly(lactide-co-glycolide)(PLGA) and *β* tricalcium phosphate (*β* TCP). A summary of the literature involving synthetic materials in combination with HA for the following sections can be found in Table 2.

*(1) Polymers*. Polycaprolactone (PCL) is a synthetic polymer which has been used in combination with HA [49]. Xia et al. produced nano-HA (nHA)/PCL scaffolds using laser sintering which were characterized for mechanical properties and tested for biocompatibility and osteogenic potential. Increasing concentrations of nHA were seen to increase hydrophilicity, osteoblast differentiation, and mineralization as demonstrated by ALP staining and Alizarin red staining. Scaffolds with the highest percentage of nHA were seen to have a slower release profile for rhBMP-2 which may indicate a tunable release profile. Therefore,* in vitro* nHA/PCL scaffolds were shown to be of potential use for bone regeneration [21]. Lu et al. created a biphasic calcium phosphate (BCP) scaffold coated with PCL and nHA and seeded primary human osteoblasts (HOBs) and ASCs. When both BCP/PCL-nHA scaffolds and BCP/PCL scaffolds were seeded with only (adipose derived stem cells) ASC cells it was observed that the HA containing scaffolds had a greater ability to induce cell spreading and gene expressions of Runx2, osteopontin, and bone sialoprotein, but osteocalcin was not upregulated in ASC cells. The MSCs were subsequently cocultured with HOB cells on BCP/PCL scaffolds and an increase in osteogenic differentiation was observed with respect to BCP/PCL scaffolds which were only seeded with ASC cells. It was also observed that the combination of a HOB coculture and a BCP/PCL-nHA scaffold displayed the largest osteogenic potential with an increase in Runx2, osteopontin, bone sialoprotein, and osteocalcin expression. Additionally, combining both modalities (coculturing HOBs on BCP/PCL-nHA scaffolds) leads to the highest increase in the gene expression of Runx2, osteopontin, bone sialoprotein, and osteocalcin. However, when HOBs were grown on the HA containing polymer higher expressions were noted. The authors indicated that the results show that coculturing with cells native to bone tissue enhanced the microenvironment of the MSCs and led to higher osteodifferentiation [22].

Nanoparticles of hydroxyapatite (nHA) are often used in combination with synthetic and natural materials to achieve a high degree of flexibility that imitates natural bone in an efficient manner. Ramier et al. created one such scaffold consisting of nHA, polyhydroxybutyrate (PHB), and gelatin that reflect the mechanical strength of bone and the osteoconductivity and osteoinductivity necessary in a bone scaffold. The use of this scaffold in combination with MSCs caters to different areas of bone regeneration applicability. The authors found that electrospinning a gelatin/PHB mixture followed by electrospraying nHA led to the formation of a rough surface morphology conducive to that of the natural bone and confirmed this with SEM visualization. This bone reflective morphology substantially increased the fibrous surface, which in turn allowed greater interaction between nHA and MSCs resulting in an increase in osteoinductivity and osteoconductivity indicated by ALP activity [23].

In another study a BCP/polyvinyl alcohol (PVA) scaffold was synthesized by Nie et al. and seeded with BMSCs. Using SEM it was observed that this scaffold had good biocompatibility and spreading with MSCs. Although the SEM analysis was not compared to control scaffolds, the scaffolds' similarities to bone porosity, mechanical strength, and MSC attachment support make BCP/PVA scaffolds useful for bone tissue engineering [24].

*(2) Copolymers*. Poly(lactide-co-glycolide) is a synthetic polymer which is fairly inexpensive and is extremely customizable. A study from Lv et al. aimed to determine if poly(D,L-lactide-co-glycolide) (PLGA)/nHA scaffolds can be used in high-aspect ratio vessel (HARV) bioreactors for MSC proliferation and differentiation to an osteogenic lineage. Through assessment of total DNA quantity it was determined that PLGA/nHA scaffolds had higher cell proliferation than PLGA-only scaffolds. Additionally, the composite scaffold showed higher ALP activity, Alizarin red staining, osteopontin staining, and osteocalcin staining than the control. These results indicate that PLGA/nHA scaffolds showed more osteodifferentiative potential than PLGA [25].

In a different study, a novel triacrylate-co-trimethylolpropane tris(PETA-co-TMPTMP)/HA synthesized by Garber et al. was evaluated in terms of mechanical stability and interactions with ASCs. The scaffolds were created in either solid or foam form for cell seeding. Both foam and solid polymers allowed the ASCs to grow; however, as shown by Alamar blue stain, they had lower metabolic activities than PETA and styrene plate controls. To account for this reduced activity the author suggests differentiation to an osteoblastic lineage and not decreased viability. Although additional osteogenic stainings were not performed it was determined that tris(PETA-co-TMPTMP)/HA scaffolds are a novel scaffold in combination with ASCs bone degeneration [26].

To determine if poly(1,8-octanediol-co-citrate)(POC)/HA polymers had potential uses for bone regeneration Chung et al. created these scaffolds through a foaming process. Scaffolds with varying percentages of HA were created and seeded with MSCs. No significant differences in attachment were seen between the scaffolds of various concentrations, but higher amounts of HA increased ALP activity. Evidence for the osteodifferentiative potential of POC/HA scaffolds is presented through their* in vitro* results [27].

### 4.2. Dual Differentiation

Furthermore, when aiming to regenerate bone tissue it is often necessary to consider the influence of the surrounding cartilage and vasculature. A summary of the literature involving* in vitro* differentiation of MSCs into osteogenic tissue and vasculogenic or chondrogenic tissue for the following sections can be found in Table 3.

#### 4.2.1. Angiogenesis with respect to Osteogenesis

Angiogenesis plays a pivotal role in the development and repair of bone tissue. The beneficial aspects of the blood vessel formation include the plentiful oxygen supply and growth factors such as vascular endothelial growth factor (VEGF) that stimulates the overall synergistic compatibility of both angiogenesis and osteogenesis. Recent research in this area has taken positive strives by applying both osteogenic precursors in the form of MSCs and vasculogenic inducing materials to the synthesis of osteogenic tissue, thus reflecting the natural state of bone formation. The research discussed below covers the novel approach mentioned above in combination with HA based scaffolds in order to maximize osteogenic capability.

In a study by Gardin et al. it was shown that in the presence of a specific differentiation medium, a HA scaffold (Orthoss) coated with fibronectin could be used as a platform for osteogenic and vasculogenic differentiation. Adipose derived mesenchymal cells (ASCs) were seeded on these scaffolds under four media conditions: osteogenic, vasculogenic, both media, and nondifferentiative media. When osteogenic medium was used gene expression showed osteogenic differentiation through a statistically significant upregulation of osteopontin, Runx2, osteocalcin, osteonectin, and collagen 1 as well as a statistically significant downregulation of peroxisome proliferator-activated factor gamma (PPAR*γ*). When the ASCs were exposed to the vasculogenic media the expression of endothelial cell markers CD31, von vW, and vascular endothelial growth factor (VEGF) was increased and the aforementioned osteogenic markers were marginally increased. Furthermore, the use of both media led to the increase of all previously stated markers. Therefore, HA/fibronectin scaffolds have the ability to stimulate the differentiation of more than one lineage based on the induction media used [28].

Leotot et al. show that coating a HA/*β*-TCP bioceramic with hPL directly contributes to an increase in cell adhesion and proliferation by hMSCs and endothelial progenitor cells. In turn, the host cells play a role in cell recruitment to the defect area through a paracrine effect. The hPL consists of several growth factors that are proosteogenic and proangiogenic and also induce MSCs seeded on the scaffold to secrete their own growth factors such as placental growth factor (PGF) and vascular endothelial growth factor (VEGF). This was found to assist in vascularization through the recruitment of endothelial cells (ECs) [29]. In a similar study, Chen et al. found that dental pulp stem cells (DPSCs) in combination with hPL seeded on a HA/*β*-TCP bioceramic lead to increased rates of proliferation and mineralized differentiation of the DPSCs as verified by ALP activity [30].

In an article by Sun et al. the efficacy of using three-dimensional silk fibroin/HA scaffolds through direct write assembly (three-dimensional printing) for bone regeneration is assessed. The MSCs were seeded on scaffolds with pore sizes ranging from 200 to 750 *μ*m and cells were seen to align along the direction of the fibers in comparison to tricalcium phosphate (TCP) controls. When osteogenic medium was applied, collagen I staining was seen to be positive, but Von Kossa and osteocalcin were negative. Human mammary microvascular endothelial cells (MMECs) were also seeded on the scaffold and the author found morphological characteristics of angiogenesis by bright field confocal microscopy. Furthermore, coculturing the two cell types on the scaffold leads to network-like vascular structure formation. The ability of silk/HA scaffolds to support differentiation of various cell types is shown although data supporting the determination of osteogenesis and angiogenesis (a key factor in osteogenesis) was neither compared against controls nor quantified. Therefore, the potential of HA/silk scaffolds for osteogenic repair is highly encouraging although, as the author acknowledges, further work is required [31].

#### 4.2.2. Chondrogenesis with respect to Osteogenesis

The significance of osteochondrogenic tissue in the development of effective bone has been well documented. The chondrogenic tissue alongside the osteogenic progenitors influences the development of a network of tissue that could serve to reinforce the structural dexterity of bone. The recent research highlighted below reflects the significance of the osteochondrogenic interaction and looks into the unique matrix formed by the combination of chondrogenic and osteogenic tissue.

Galperin et al. achieved the coculture of chondrocytes and hMSCs by generating a bilayered scaffold constructed from two different materials: methacrylated hyaluronic acid (HAcMA) and methacrylated hydroxyapatite (HApMA). The scaffolds were generated with an innovative pore control system, which allowed for the definition of optimal pore size on a tissue specific basis. In this way, a 38 *μ*m pore size was chosen for the HApMA onto which hMSCs were seeded and 200 *μ*m pore size for the HAcMA seeded with chondrocytes. The presence of the polyhydroxyethyl methacrylate (pHEMA) was intended to serve as a sacrificial layer, coalescing the chondrogenic and osteogenic layers as the pHEMA degraded. After four weeks of culture, the bilayered scaffold showed that the hMSCs in the HApMA had formed a complete, continuous network throughout the pores, mineralizing the walls of the scaffold. Furthermore, the walls of the scaffold stained positive for Alizarin red even after comparison to an acellular HApMA control. This result indicated that calcium was indeed present, not due to the initial HA, and confirms the osteoinductive influence of HA on the hMSCs. The chondrocytes on the HAcMA scaffold were also successful in generating a developed ECM, similar to native cartilage due to the chondroconductive nature of hyaluronic acid, as well as the optimal pore size. Further, degradation of the pHEMA allowed for the integration of the layers and successive success of the osteochondral engineered scaffold [32].

An additional study performed by Zhou et al. also highlights the importance of collagen/HA scaffolds. In this study collagen scaffolds containing a gradient of HA (such that the bottom layer had a high concentration of HA while the top had little to none) were synthesized using a freeze-drying method and studied for their potential to form interfacial tissues. Specifically, the ability of the scaffold to induce bone marrow MSCs (BMSCs) to differentiate into osteoblasts or chondrocytes based on their location on the scaffold was determined. Using alcian blue and collagen II staining as well as glycosaminoglycan (GAG) quantification and qRT-PCR for osteogenic markers the author found that chondrogenic differentiation was more prevalent in the scaffold location with low HA. Conversely, the scaffold location that contained the highest level of HA was seen to promote osteogenic differentiation. Additionally, from protein and calcium staining, enzyme activity, and gene expression, it was determined that the side of the scaffold with high HA was more osteogenic than either the side of the scaffold with low HA or a HA control. These results indicate that the combination of HA and collagen is more osteoconductive than low HA/collagen or HA control scaffolds and represents an example of how HA's osteogenic properties can be enhanced by adding natural materials. However (as the authors acknowledge) chondrogenic and osteogenic formation required the use of differentiation mediums and were done separately and superficially. To perform both differentiation procedures on one scaffold the author suggests the use of a double-chambered stirred bioreactor and to increase cell infiltration the author proposes the use of a leak proof collagen sponge [33].

## 5. Recent Advances in Skeletal Disease/Injury Treatment

The wide applicability of the genesis of osteogenic tissue* in vitro* becomes apparent when understating a variety of physiological contexts in which bone defects arise. It is important to note that the treatment of bone defects directly depends on characteristics of the defect, therefore leading to different methods of osteoreparation. For example, complete fractures of long bones may require employment of fixative agents in order to immobilize the area and facilitate healing, while void-like defects require a filling material that can promote bone formation when the injury has reached a critical size. The majority of these solutions, be it via filling materials, fixation agents, or else, capitalize on the production of new bone for successful solution of the symptoms, and thus all part from the same starting point: the generation of bone-precursing osteoids. The relevance of generating osteoids as a promising first step to bone formation has led to the design of studies that strive to achieve their formation by directly applying HA* in vivo*. A summary of the literature involving* in vivo* treatment of skeletal disease/injury using HA containing scaffolds and MSCs can be found in Table 4.

Having established these goals, a recent study conducted by Wang et al. used human umbilical cord mesenchymal stem cells (hUCMSCs) in combination with a nHA, chitosan, and PLGA scaffold. Along with an increase in ALP activity and osteocalcin the results concluded that attachment, proliferation, and osteogenic differentiation of the hUCMSCs were best noted in the trimodality scaffold. This scaffold, therefore, provided a dynamic biodegradable and osteoinductive scaffold for bone regeneration. Furthermore, Wang et al. showed through H&E staining that subcutaneous additions of nHA/CS/PLGA scaffolds seeded with hUCMSCs resulted in a statistically significant increase in osteoid tissue formation [34]. In a similar manner Leotot et al. subcutaneously implanted HA/Beta-TCP scaffold seeded with hMSCs and performed immunohistochemistry of the implantations. It was discovered that, by coating the surface of the scaffold with hPL, a higher degree of osteogenic regeneration and angiogenesis could be achieved [29]. In relation to this is an experiment carried out by Chen et al. where they assessed the dose of hPL in subcutaneously implanted HA/Beta-TCP scaffolds seeded with MSC type stem cells. They found that the concentration of PL (which was approximately 5%) heavily influences the proliferation and mineralization of the stem cells and tissue regeneration [30].

### 5.1. Tumor Resections

Neoplasm formations on bone can lead to severe deformation and progressive loss of mechanical integrity and support. Standard treatment of these abnormal tissues includes surgical resection via debridement to preserve healthy tissue [50]. In this way, although the treatment intends to avoid further deformations because of the growth of the neoplasm, they usually result in gaping voids in the bone. If these induced bone defects are too large, their critical size makes natural regeneration of the area improbable. For this reason, engineered tissue constructs containing hMSCs have been proposed to be a possible means of forcibly inducing osteoregeneration. In this line of reasoning, Fu et al. recently used HA with calcium carbonate to increase the degradation rate of the scaffold so that it could appropriately degrade and stimulate bone growth in a void. Coralline HA/calcium carbonate (CHACC) scaffolds were created by partial conversion of coralline calcium carbonate to hydroxyapatite. These scaffolds were then characterized* in vitro*, tested* in vivo*, and implemented in a clinical trial for their potential use in bone regeneration. For the* in vitro* experiments, cells were either cultured on glass slides or CHACC scaffolds with or without osteogenic media. At first cells cultured on glass slides proliferated more quickly, but both reached confluence after 16 days. MSCs on CHACC scaffolds showed more ALP activity and cell specific ALP activity than those on glass slides and the use of osteogenic media appeared to deposit more mature collagen. Although data for CHACC scaffolds was not compared to HA scaffolds this paper shows that CHACC scaffolds have osteogenic potential, especially when used in combination with osteogenic media. Fu et al. then investigated the potential for bone regeneration with coralline hydroxyapatite/calcium carbonate (CHACC) seeded with hMSCs* in vivo* to assess the osteogenic potential in immunodeficient mice. The CHACC implanted subcutaneously on the dorsal surface was examined 10 weeks after surgery with SEM. The CHACC without MSCs resulted in minimal fibrous tissue formation with no bone formation. The CHACC with MSCs induced bone formation on the surface of the scaffold as seen with SEM. Prior to implantation risedronate was used to inhibit resorption of the scaffold. The CHACC scaffold alone then underwent clinical scrutinization in an attempt to induce bone expansion after tumor removal. Successful regeneration in 16 patients occurred after 4 months on average. These results are promising; however, coral's abundance could be a problematic issue due to its limited availability [35].

### 5.2. Cranial Facial Defects

Craniofacial defects are characterized as abnormal development of the cranial bones during gestation as a result of genetic or environmental factors or a combination of the two. The most common craniofacial birth defects in humans are collectively known as orofacial clefts, of which the most common are cleft lip and palate [36]. Restructuring of the hard oral palate is commonly performed via autologous bone grafts; however, the pervasiveness of postsurgical suffering and the high occurrence of oronasal fistula [51] result in the need for a better alternative. Further, it is important to consider that because these abnormalities arise in infants (due to their congenital nature), autologous grafting may also deeply affect both proper autologous graft sourcing and the proper functioning of the graft source tissue. The aforementioned postsurgical suffering compounded with the issue of efficient sourcing makes tissue engineered bone an attractive solution. Currently, the nature of the condition and the type of patient will also restrict the way these must be designed since many of these scaffolds will have to adapt to the rapidly changing tissue architecture that occurs with normal child development.

A clinical case by Stanko et al. reports the use of MSCs on a collagen membrane based scaffold in combination with platelet gel (consisting of HA particles and PRP coagulated with the use of calcium ions) to treat a patient with an oronasal fistula (ONF) in the alveolar cleft [36]. MSCs extracted from the patient's bone marrow were seeded onto the collagen membrane 3 weeks preceding the surgery and during the surgery the membrane-cell combination was placed within the wound. The platelet gel membrane was synthesized from 2 grams of HA (0.5 mm) particles and 1.5 mL of PRP with 10% calcium gluconate to induce coagulation. The wound was filled with the gel and additional MSCs and PRP. After ten weeks bone formation at the site of the ONF was visible. An alternative method to treat alveolar cleft defects was carried out by Behnia et al. where a trimodality scaffold consisting of biphasic (HA/TCP), MSCs, and platelet-derived growth factor (PDGF) was utilized in 3 patients with the defects [37]. MSCs extracted from the bone marrow of the patients were seeded on the biphasic scaffold 1 day prior to implantation. Before the surgery, the PDGF was added to complete the trimodality scaffold and the scaffold was implanted in the defect. After 3 months, the alveolar premaxillary clefts were examined in the three patients and a mean of 51.3% bone regeneration was reported. The osteogenic potential of this method was less than expected and less than achieved with other experiments that used rhBMP-2 or autogenous iliac graft [52]. Additionally, the lack of a control group further weakened the results.

Demonstrating the use of a multimaterial scaffold consisting of 60% HA and 40% *β*-TCP, Jazedje et al. treated nonimmunosuppressed (NIS) rats for cranial defects [38]. This experiment combined the bioceramic scaffold with MSCs derived from the Human Fallopian Tube (htMSC) to investigate bone regeneration with this unique source of stem cells. When comparing the histological analysis between a cranial defect in the left side treated with the bioceramic alone and the right side treated with the bioceramic and the htMSC, neobone formation and mature bone formation occurred at a more rapid pace and were more substantial in development in the bioceramic-htMSC treated defect. Besides the osteogenic potential of the MSCs, less inflammatory response occurred; this aligns with previous research that has shown the potential of MSCs to decrease inflammation [53]. Yang et al. examined the effects of the drug strontium on bone formation in rats with calvarial defect via the use of collagen-strontium-substituted hydroxyapatite (collagen-Sr-HA) scaffolds. At both one- and three-month intervals the progress was noted. At each interval the bone density increased substantially in comparison to a scaffold of collagen and a scaffold of collagen-HA. After 3 months the collagen-Sr-HA group of rats showed complete regeneration of the defect area giving rise to a 11.6 ± 0.6-fold increase in mature bone formation compared to 3.4 ± 0.7 in the HA group. Furthermore, extracellular matrix (ECM) increased at a greater rate in the collagen-HA scaffold than in that of the other groups as shown in increasing levels of collagen. The strontium also upregulated Beta-catenin expression levels* in vivo* which contributes to greater osteoblastic differentiation and further bone regeneration [39]. In a similar setting, Gardin et al. also inflicted a calvarial defect in a set of 24 rats to examine engraftment of tissue engineered bone grafts [28]. Two calvarial defects were inscribed per animal where one was a control treated with a HA scaffold and the other was treated with an adipose derived stem cell (ASC) seeded HA scaffold. An inflammatory response resulted in the HA implant while no inflammatory response occurred with the HA-ASC scaffold. Also, collagen type I, osteopontin, osteonectin, osteocalcin, and RUNX2 were higher in the HA-ASC implant signifying osteogenesis and ECM formation. The fibroblast-like cells visible in the granules of the HA were responsible for both the osteogenic markers expressed as well as the vessels that were visible within the HA.

### 5.3. Load Bearing Defects

Fractures are a significant source of bone defects that can result from the blunt trauma delivered to skeletal tissue during traumatic accidents, as a consequence of chronic demineralization like the case of osteoporosis, improper biomechanics during natural gait, and so forth. Treating these requires a different approach to void-based bone defects considering the biomechanical nature and geometry of the lesion. Taking into account the region in which the injury is located is a chief concern, especially when one considers cases in which there are loading constraints on a scaffold, such as in femoral and vertebral defects. Tissue construct based treatments therefore require redefining important physiological parameters of scaffolds designed to encase the delivered cell phase in order to ensure effective osteoregeneration.

A comparative project was performed by Amorosa et al. to examine critical sized segmental defects in the femur of rat models [40]. The four different variables looked at were autographs, allographs, the polymer based scaffold of poly-*ε*-caprolactone, and hydroxyapatite with and without MSCs. Radiography showed that callous formation was more significant in the allograph and autograph. While the polymer based scaffold had less callous formation, the scaffold with MSCs had more callous formation than without the MSCs. Biomechanical testing compared the femoral repair method to that of its respective contralateral control. The scaffold alone repair resulted in a dramatic decrease in the elastic stiffness and viscous stiffness in comparison to the other groups and the contralateral control. However, adding MSCs to the polymer based scaffold contributed to an increase in elastic stiffness and viscous stiffness and a decrease in phase angles. This demonstrates that the MSCs facilitate the repair of a more bone-like biomechanical structure. The MSCs also contribute to greater bone generation as can be seen with the callous formation. This study proves the efficacy of the MSC seeded poly-*ε*-caprolactone and hydroxyapatite scaffold as a possible alternative to allografts and autografts for critical segmental defects. It is worth noting that a low amount of samples and limited biomechanical testing limit the project applicability as a whole.

Research into vertebral body fractures remains a focus in research due to the fact that these inflicting fractures remain one of the most common injuries in individuals. Vaněček et al. investigated the therapeutic applicability of MSCs seeded on HA to treat vertebral defects. The four groups compared were the HA scaffold alone, the HA with 500 k MSCs, HA with 5 million MSCs, and a control consisting of the HA scaffold with no MSCs. Both micro-CT scans and histological examinations found that the HA scaffold seeded with 5 million MSCs showed positive results compared to the others. The inflammation was negligible and the osteoinductivity was remarkably higher than that with the scaffold alone, which is evident from the increase in bone formation [41].

## 6. Conclusions

As previously discussed, HA is most often used for bone regeneration and osteodifferentiation due to its osteoconductive and osteoinductive properties. HA is found in high quantities in native bone and when used in the body leads to a nonimmunogenic response reinforcing the applicability as a biocompatible osteogenic solution. Furthermore, when used in nanoparticle form, HA can significantly enhance the fibrous morphology of a material which influences cell proliferation and differentiation.

Although HA is useful for the osteostimulation of bone, the choice of additional materials for fabrication often has a defining effect on the function of the scaffold. Natural materials often entail a certain level of immunoinertness and biodegradability and can be included in scaffolds for differentiative purposes. Conversely, synthetic materials are modifiable and often mass producible, a desirable trait when considering scale-up for various patients and/or large defect areas. By combining synthetic and natural materials, the benefits of each can be combined into a single scaffold.

As highlighted in this review, combining stem cells, in particular, MSCs, into the various HA based scaffolds increases the scaffolds potential use for bone regeneration. Adding the benefits of MSCs immunomodulatory, immune-inert, and immune-privileged state to a synthetically or naturally enhanced HA scaffold has demonstrated superior results than the scaffolds alone.

Future work with MSCs seeded on HA containing scaffolds appears to be heading toward the incorporation of near bone tissues. In recent studies dual sided osteochondral grafts have been created for use in diseases affecting bone and chondral tissue [33]. Additionally, scaffolds are often used to promote angiogenesis because osteogenesis has been seen to be reliant on vascular tissue.

In conclusion, HA is a material which most often induces osteogenesis both* in vivo* and* in vitro*, although the production of vascular tissue has been seen. Adding HA to other materials (either natural or synthetic) could, therefore, modulate the osteogenic potential and mechanical properties of the subsequent mixture. Furthermore, MSCs can be included in such scaffolds for differentiation to osteogenic lineages and/or implantation for bone defects purposes although differentiation media are often required. Therefore, HA scaffolds containing MSCs can be used as a combinatorial modality for treating bone disease and degeneration.

## Figures and Tables

**Table 1:** Summary of references for natural materials in combination with hydroxyapatite.

Source of stem cells	Material used	Results	Study/reference
hMSCs	Collagen/fibronectin/HA	Cells are viable on the scaffold	Antebi et al. 2013/[12]
BMSCs and DPSCs	Fibronectin/collagen/albumin coating for HA versus allographs	↑ cell attachment for allographs coated with albumin	Weszl et al. 2012/[13]
BMSCs, PDL fibroblasts, and HBCs	Gelatin/HA	↑ ALP activity for moderate HA levels	Rungsiyanont et al. 2012/[14]
WJ-MSCs	HA/gellan gum/gelatin	Cells are viable on the scaffold	Barbani et al. 2012/[15]
BMSCs	CS/HA	↑ osteocalcin expression/staining, ↑ ALP expression/staining, ↑ Col1*α*I expression, ↑ Runx2 expression	Kim et al. 2013/[16]
BMSCs	CS/hyaluronic acid/nHA	↑ ALP activity	Chen et al. 2012/[17]
BMSCs	CS/fibronectin/vitronectin/nHA	↑ calcium deposition, ↑ collagen content, ↑ total protein synthesis	Wang et al. 2014/[18]
hMSCs	CS/PgA/nanoclay	↑ ARZ staining, ↑ ALP activity	Ambre et al. 2013/[19]
BMSs	Silk/HA	↑ collagen I staining, ↑ bone sialoprotein staining, ↑ osteocalcin staining, ↑ calcium deposition	Bhumiratana et al. 2011/[20]

**Table 2:** Summary of references for synthetic materials in combination with hydroxyapatite.

Source of stem cells	Material used	Results	Study/reference
BMSCs	PCL/nHA	↑ ALP staining, ↑ Alizarin red staining, ↑ rhBMP-2	Xia et al. 2013/[21]
Primary human osteoblasts/ASCs	PCL/BCP-nHA	↑ Runx2 expression, ↑ osteopontin expression, ↑ bone sialoprotein expression, ↑ osteocalcin expression	Lu et al. 2012/[22]
(WJ) MSCs	PHB/gelatin/nHA	↑ ALP activity	Ramier et al. 2014/[23]
BMSCs	PVA/BCP	Favorable morphological characteristics	Nie et al. 2012/[24]
BMSCs	PLGA/nHA	↑ ALP activity, ↑ Alizarin red staining, ↑ osteopontin staining, ↑ osteocalcin staining	Lv et al. 2013/[25]
ASCs	Tris(PETA-co-TMPTMP)/HA	↓ Almar blue staining	Garber et al. 2013/[26]
BMSCs	POC/HA	↑ ALP activity	Chung et al. 2012/[27]

**Table 3:** Summary of references for dual differentiation.

Source of stem cells	Material used	Results	Study/reference
ASCs	Fibronectin/HA	↑ osteopontin, ↑ Runx2, ↑ osteocalcin, ↑ osteonectin, ↑ collagen 1, ↓ peroxisome proliferator-activated factor gamma	Gardin et al. 2012/[28]
hMSC	HA/Beta-TCP with hPL coating	↑ PGF, ↑ VEGF, ↑ ALP Activity	Leotot et al. 2013/[29]
hMSC	HA/Beta-TCP with hPL media	↑ mineralization	Chen et al. 2012/[30]
MMEC, BMSCs	Silk/HA	MSC only: ↑ collagen I staining, Von Kossa staining, osteocalcin staining Coculture: vascular network-like formation	Sun et al. 2012/[31]
Chondrocytes/hMSCs	Methacrylated hyaluronic acid/methacrylated hydroxyapatite	Positive calcification staining and extracellular matrix development	Galperin et al. 2013/[32]
BMSCs	Collagen/HA	High HA/collagen is more osteogenic while low HA/collagen induces chondrogenic differentiation	Zhou et al. 2011/[33]

**Table 4:** Summary of references for recent advances in skeletal disease/injury treatment.

Source of stem cells	Material used	Results	Study/reference
hUCMSCs	CS/PLGA/nHA	↑ osteocalcin, ↑ ALP activity, ↑ osteoid tissue formation	Wang et al. 2014/[34]
hMSC	HA/Beta-TCP with hPL coating	↑ osteogenic regeneration, ↑ angiogenesis	Leotot et al. 2013/[29]
hMSC	HA/Beta-TCP/hPL/HA	↑ mineralization	Chen et al. 2012/[30]
BMSCs	CHACC	↑ ALP activity, ↑ mature collagen deposition, and bone tissue formation	Fu et al. 2013/[35]
BMSCs	Collagen/platelet gel	Bone formation was visible after ten weeks	Stanko et al. 2013/[36]
BMSCs	TCP/PDGF/HA	Healing without complications	Behnia et al. 2012/[37]
htMSC	Bioceramic	↑ neobone formation, ↓ inflammation	Jazedje et al. 2012/[38]
hUCMSCs	Collagen/Sr/HA	↑ bone density, ↑ bone formation, ↑ ECM formation, ↑ Beta-catenin	Yang et al. 2011/[39]
ASCs	HA	↑ RUNX2 expression, ↑ osteopontin expression, ↑ osteocalcin expression, ↑ expression staining	Gardin et al. 2012/[28]
BMSCs	Autograph, allograph, PCL/HA	↑ elastic stiffness, ↑ viscous stiffness, ↑ callous formation	Amorosa et al. 2013/[40]
BMSCs	HA	↑ osteoinductivity, ↓ inflammation	Vaněček et al. 2013/[41]
